# Programmatic conversion of crystal structures into 3D printable files using Jmol

**DOI:** 10.1186/s13321-016-0181-z

**Published:** 2016-11-23

**Authors:** Vincent F. Scalfani, Antony J. Williams, Valery Tkachenko, Karen Karapetyan, Alexey Pshenichnov, Robert M. Hanson, Jahred M. Liddie, Jason E. Bara

**Affiliations:** 1 University Libraries, Rodgers Library for Science and Engineering, The University of Alabama, Tuscaloosa, AL 35487 USA; 2 ChemConnector, 513 Chestnut Grove Court, Wake Forest, NC 27587 USA; 3 Royal Society of Chemistry, 14900 Forest Landing Circle, Rockville, MD 20850 USA; 4 Department of Chemistry, St. Olaf College, Northfield, MN 55057 USA; 5 Department of Chemical and Biological Engineering, The University of Alabama, Tuscaloosa, AL 35487 USA

**Keywords:** 3D printing, Dataset, Jmol, JSmol, Crystals, Crystallography, Visualization, Open data

## Abstract

**Background:**

Three-dimensional (3D) printed crystal structures are useful for chemistry teaching and research. Current manual methods of converting crystal structures into 3D printable files are time-consuming and tedious. To overcome this limitation, we developed a programmatic method that allows for facile conversion of thousands of crystal structures directly into 3D printable files.

**Results:**

A collection of over 30,000 crystal structures in crystallographic information file (CIF) format from the Crystallography Open Database (COD) were programmatically converted into 3D printable files (VRML format) using Jmol scripting. The resulting data file conversion of the 30,000 CIFs proceeded as expected, however some inconsistencies and unintended results were observed with co-crystallized structures, racemic mixtures, and structures with large counterions that led to 3D printable files not containing the desired chemical structure. Potential solutions to these challenges are considered and discussed. Further, a searchable Jmol 3D Print website was created that allows users to both discover the 3D file dataset created in this work and create custom 3D printable files for any structure in the COD.

**Conclusions:**

Over 30,000 crystal structures were programmatically converted into 3D printable files, allowing users to have quick access to a sizable collection of 3D printable crystal structures. Further, any crystal structure (>350,000) in the COD can now be conveniently converted into 3D printable file formats using the Jmol 3D Print website created in this work. The 3D Print website also allows users to convert their own CIFs into 3D printable files. 3D file data, scripts, and the Jmol 3D Print website are provided openly to the community in an effort to promote discovery and use of 3D printable crystal structures. The 3D file dataset and Jmol 3D Print website will find wide use with researchers and educators seeking to 3D print chemical structures, while the scripts will be useful for programmatically converting large database collections of crystal structures into 3D printable files.

## Background

Three-dimensional (3D) printed crystal structures offer several advantages over traditional molecular model constructs (e.g. plastic molecular model kits, Styrofoam balls, beads). Perhaps the greatest advantage is that 3D printing is capable of fabricating extremely complex molecular structures that would be difficult or impossible to create with traditional molecular model fabrication techniques [1]. As such, numerous researchers have recently used 3D printed crystal structures to advance their teaching and research [1–7].

Digital 3D printable crystal structure files can be manually created from standard crystallographic information files (CIF) using a variety of open source, free, and commercial software packages [1, 2, 4–6]. In general, the crystal structure is first opened in a crystallographic viewing program [8–12] where the structure representation is adjusted to preference. For example, it may be necessary to display the atoms as spheres, delete unwanted molecules, or generate a unit cell. Once the desired crystal structure representation is achieved, the structure is typically exported as either a stereolithography file (STL) for single color 3D printing or a virtual reality modeling language file (VRML) for multi-color printing. Lastly, files may need to be repaired of any inconsistencies in the surface of the 3D model (e.g. holes, overlaps) and verified for compatibility with 3D printer software before printing. In short, manual conversion of crystal structures into 3D printable file formats can be a tedious and time consuming process. As there are hundreds of thousands of crystal structures currently available in open access databases such as the Crystallography Open Database (COD) [13, 14], it would be ideal to automate the conversion of crystal structures (CIFs) from these databases into 3D printable file formats. Providing quick access to 3D printable crystal structure files removes the barriers associated with file manipulation and conversion, which makes it easier for educators and researchers to 3D print crystal structures.

There are currently only two methods available for programmatic conversion of a crystal structure into a 3D printable file including the NIH 3D Print Exchange web tools developed by Coakley and coworkers at the NIH [15–17] and the stand-alone Cif2VRML Windows application developed by Kaminsky [18]. The NIH 3D Print Exchange web tool uses Chimera [12] and Blender [19] scripts that automatically process crystal structures into 3D printable files. The Cif2VRML application is written in the Delphi programming language and allows for one-click creation of 3D printable files from CIFs. The NIH 3D Print Exchange web tool and Cif2VRML application have greatly advanced the process and efficiency of creating 3D printable crystal structures. However, crystal structures are still converted on an individual basis; users must upload or process each crystal structure individually. The NIH 3D Print Exchange Team has made all scripting code available in the public domain, so researchers could adapt these scripts to programmatically process multiple files locally in the future [16]. Multiple file processing is also not currently available in the Cif2VRML application, but is being considered in a future release [18].

In this article, we report our progress to overcome the current limitations with 3D printable crystal structures; that is, the lack of programmatic multiple file conversion methods. We used a Jmol [11, 20] script to programmatically convert over 30,000 crystal structures into 3D printable files. The 3D printable file datasets and programmatic scripts are provided openly to the community on figshare. A Jmol 3D Print website was created based on the COD search interface that allows users to discover the 3D printable crystal structure file dataset via text, SMILES, reference, elements, and cell parameters. In addition, users can create a custom 3D printable file (STL or VRML) from their own CIFs and for any structure in the COD using the JSmol implementation within the 3D Print website.

## Methods

Programmatic processing of crystal structures into 3D printable files presents several challenges. One such challenge is that many crystal structures contain counterions and solvent molecules. These companion molecules would ideally be removed from the 3D printable file for two reasons: (1) most often the counterions and solvent molecules are not of direct interest for 3D printing; and (2) 3D files with multiple independent objects can be difficult for 3D printers to fabricate. While removal of counter ions and solvent molecules can be readily achieved with cheminformatics toolkits [21–23], this procedure has not yet been applied in the preparation of small molecule and extended solid 3D printable crystal structure files.

Another challenge with processing crystal structures into 3D printable files arises with the initial representation of the crystal structure within crystallographic viewers. By default, many crystallographic viewing programs only load the unique atom positions in the crystallographic data, namely the asymmetric unit. This can lead to displaying only a fraction of a molecule or a very small segment of an extended solid. To gain a complete representation of a molecule or a complete unit cell of an extended solid, the software must be instructed to apply the appropriate space group symmetry operations to the asymmetric unit [24]. While the asymmetric unit is a useful representation, we suspect in most cases, those interested in 3D printing crystal structures would prefer the symmetry operations applied to the crystallographic data. Applying the symmetry operations ensures that the representation will present either one or more whole molecules or a complete unit cell fragment of an extended solid. Moreover, with extended solids it is often useful to pack the unit cell; that is, display all atoms and bonds that fit only within one or more unit cells.

**Fig. 1:**
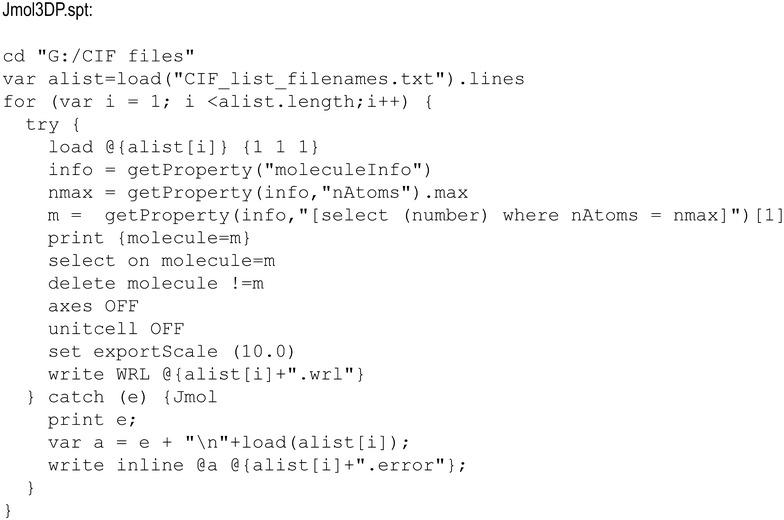
The Jmol3DP script can be used to batch process CIFs into 3D printable VRML files. The script was executed by creating a windows batch file, Jmol.bat (java -Xmx1024m -jar “JmolData.jar” -o  %1 %2 %3 %4 %5 %6 %7 %) and a text list of the crystal structure file names. Then the batch file was run from a DOS command line (Jmol –s Jmol3DP.spt). For extended solids, symmetry and packing was applied upon initial loading (i.e. edit line 5 of the script to read load @{alist[i]} {1 1 1} PACKED)

To overcome the aforementioned challenges of processing crystal structures into 3D printable files, we developed a Jmol script, Jmol3DP (Fig. 1). When the Jmol3DP script is executed with a batch file from a DOS command line, the script will automatically process any number of CIFs into VRML files using the following method:

1. A CIF crystal structure from a text file list is loaded in Jmol as a ball and stick representation and the symmetry elements appropriate to the space group are applied. Note: For extended solids, the Jmol loading option PACKED was added (i.e. load @{alist[i]} {1 1 1} PACKED). If processing biomolecules, the Jmol STRUTS command should be used to add necessary mechanical support to the model.
2. The molecule (or extended solid network) with the greatest number of atoms within the displayed structure(s) is selected.
3. All other molecules are deleted (i.e. duplicate molecules with same number of atoms and molecules with fewer atoms).
4. All axes and unit cell guides are deleted.
5. The largest molecule (or extended solid network) that was selected in step (2) is scaled ten times its original size and saved as a VRML file.
6. Steps (1)–(5) are repeated for the next CIF.

The ball and stick representation was selected as it one of the most popular visual representations of chemical structures. Scaling was necessary to generate structures with dimensions on the order of 1–10 cm length scales (in each x, y, z, axes), which are appropriate for most 3D printers.

To test our methods, a subset of CIFs within the COD were selected. This included 31,239 CIFs originating from a dataset of the COD that was also incorporated into ChemSpider [25]. This collection of CIFs was a convenient and sizable dataset composed of small organic molecules and inorganic extended solids. All CIF data within the COD is in the public domain and therefore allowed us to reuse, adapt, and share the 3D printable files created from COD data [13].

After processing the CIFs into VRML files using Jmol scripting, additional file preparation steps were added to increase the compatibility of the 3D files across numerous 3D printer software applications and 3D printing services. AccuTrans 3D (v2014-1-0) [26] was used to remove high level objects (cone, sphere, and cylinders) from the VRML files and to convert a subset of the files (11,732) into STL format. Lastly, the STL files were run through an extended batch repair process using Netfabb Professional [27]. Both AccuTrans 3D and Netfabb Professional have intuitive graphical user interfaces for batch processing.

For the 3D test prints, we used a Stratasys uPrint SE with ABS P430 XL Ivory model material and SR-30 XL soluble support material. Catalyst EX software was used to create the toolpath. Settings included SMART support structure and high density fill. Approximate print time was 4 h for each 3D printed crystal structure. The SR-30 XL soluble support material was removed with a solution of P400-SC Waterworks Soluble Concentrate (a caustic basic solution). The 3D printed crystal structures containing the associated support material were submerged in the P400-SC basic solution for 3 h at 80 °C to selectively dissolve the support material. The completed 3D printed crystal structures absent of any support material were then rinsed three times with water.

## Results and discussion

The Jmol3DP script described here was successfully used to programmatically convert CIFs from the COD into 31,239 printable VRML files and 11,732 printable STL files. Our original process involved post-processing of VRML files created by Jmol 14.1 using AccuTrans 3D and Netfabb Professional. This post-processing was necessary in order to remove the high level VRML 2.0 objects (cone, cylinder, sphere) [28] that Jmol 14.1 created and to convert the VRML to STL format, which many 3D printers require. In the process of review of this manuscript, Jmol was developed further, and Jmol 14.6.4 now exports VRML, X3D (the XML version of VRML), and STL files that can be submitted directly to 3D print services and 3D printer software without the need for any additional post-processing.

We note that success of STL and VRML files for 3D printing relies upon careful attention to details. All rendered parts must be closed surfaces—no open ends of cylinders used for bonds; no missing triangles in the geodesics used for atoms. The counter-clockwise winding of all triangles must be designed to project all triangle normals in the proper direction. Infinitely thin surfaces (which appear properly on screens and in images) must be given some thickness. Jmol’s (v14.6.4) STL and VRML exporters take care of all of this automatically. In addition, in Jmol, care has been taken to ensure that no two identical objects are placed in the same location. For example, where two bonds come together, the geodesics used to form the rounded endcaps of the cylinders are only generated once, not twice. Some more advanced features of Jmol, such as slabbed (unclosed) isosurfaces, dot surfaces, and labels, are not supported due to these restrictions.

**Fig. 2:**
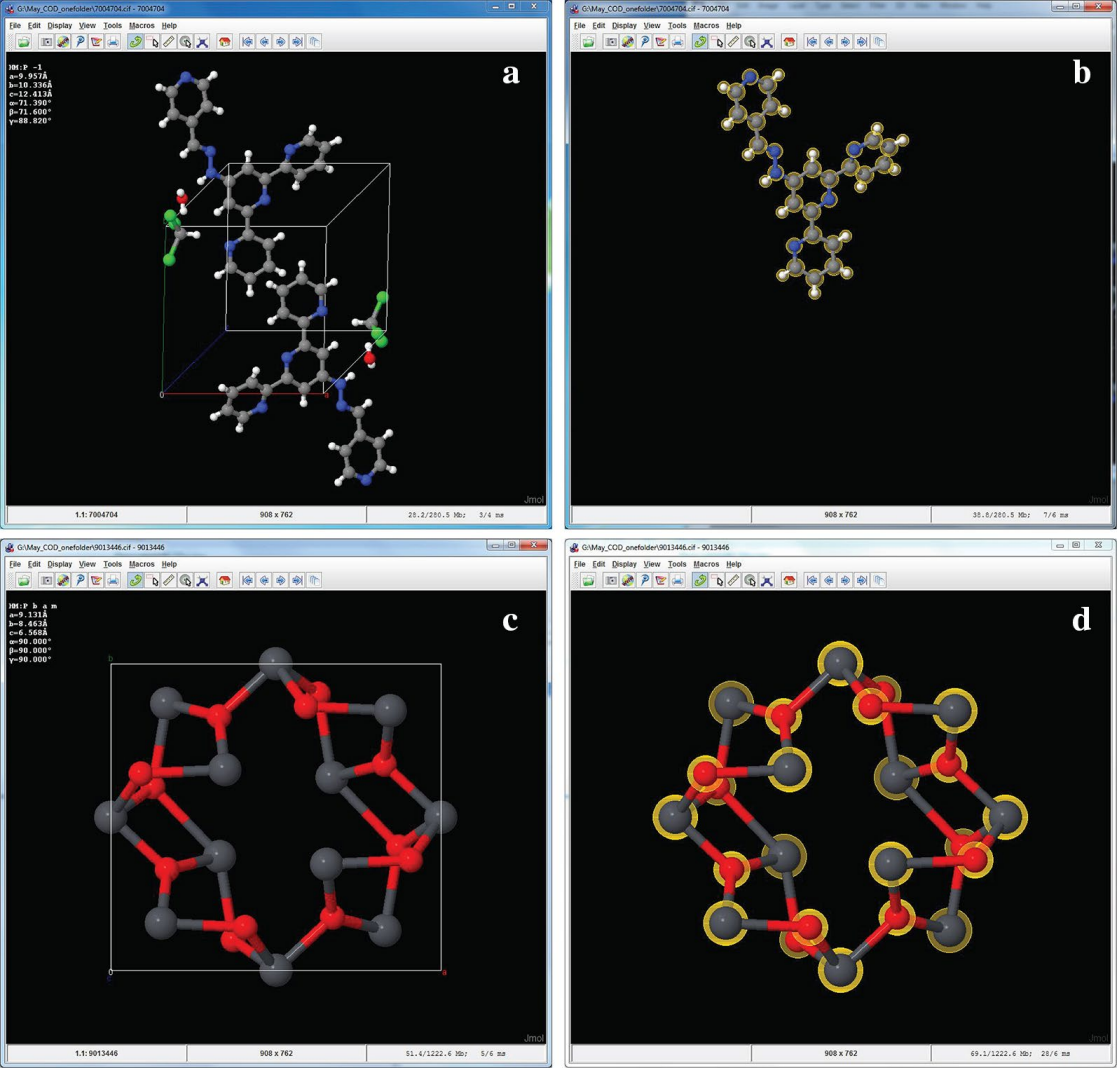
Examples of CIFs processed through the Jmol3DP script. 4′-[(2E)-2-(4-Pyridinylmethylene)hydrazino]-2,2′:6′,2″-terpyridine (COD ID: 7004704) as viewed initially in Jmol with symmetry operations applied (load 7004704.cif {1 1 1}) (**a**); 4′-[(2E)-2-(4-Pyridinylmethylene)hydrazino]-2,2′:6′,2″-terpyridine after processing with Jmol3DP script (**b**); Pb_3_O_4_ (COD ID: 9013446) as viewed initially in Jmol with symmetry operations and packing applied (load 9013446.cif {1 1 1} PACKED) (**c**); Pb_3_O_4_ after processing with Jmol3DP script (**d**)

Two representative examples of CIFs processed with the Jmol3DP script are shown in Fig. 2. The crystal structure of 4′-[(2E)-2-(4-Pyridinylmethylene)hydrazino]-2,2′:6′,2″-terpyridine viewed in Jmol, as loaded with the symmetry operations applied, contains two identical molecules of the pyridinyl terpyridine derivative (differing in orientation), two chloroform molecules, and two water molecules (Fig. 2a). After running the Jmol3DP script, only one pyridinyl terpyridine molecule is selected, scaled, and exported as a VRML file. All other molecules and axes were automatically deleted (Fig. 2b). In the extended solid example, Pb_3_O_4_, the crystal structure is loaded in Jmol with symmetry and packing applied (Fig. 2c). After running the Jmol3DP script, the entire Pb_3_O_4_ packed structure is selected, scaled, and exported as a VRML file; the axes and unit cells were automatically deleted (Fig. 2d). In both examples, a VRML 3D model file was created containing only the single desired chemical structure.

The quality of the programmatically converted 3D printable crystal data is satisfactory. We manually verified a random set of 115 3D printable files and found 90% accuracy. Accuracy here is being defined as producing the expected digital 3D printable molecule or extended solid from the original CIF. The Jmol3DP script automatically deleted small solvent and counterions, and ultimately produced a 3D printable file containing a single molecule or extended solid with high fidelity to the original selected structure within the crystallographic data. Inaccuracies and undesired results in the data mostly arose from co-crystallized structures where both molecules could be of interest for 3D printing. With our current 3D printing conversion method, only the larger molecule would be available as a 3D printable file, and as a result, the smaller co-crystallized molecule is deleted. A similar situation occurred with large or complex counterions. The molecule of interest contained fewer atoms than its counterion, and as a result was mistakenly deleted. Lastly, racemic mixtures present a further complication for processing. Enantiomers contain the same number of atoms. With the current Jmol3DP script, only one of the enantiomers would be selected at random. At this time it is not clear how to circumvent these challenges with programmatic multi-file processing of crystal structures for 3D printing. One method may be to write a Jmol script that produces a separate 3D printable file for each molecule in (or partly in) the crystal structure unit cell. This would preserve *all* structures in the original CIF and allow the end-user to select the molecule/extended solid of most interest. Until such a method is developed, it will be necessary for the community to help manually curate 3D printable crystal structure data.

Tests with the NIH 3D Print Exchange web tools [15] confirmed that its process, in contrast to ours, retains all molecular structures in the CIF data, makes no attempt to remove solvent molecules or counterions from small molecules and extended solids, does not apply symmetry, and does not select a defined subset for representation. If a user wishes to selectively 3D print molecules or atoms, that selection must be done manually before uploading to the system. Similarly, the Cif2VRML application does not remove solvent molecules (except for biomolecules) or counterions, does not apply symmetry, and does not automatically select a particular structure for representation. Several of these options are reported to be included in a future release of the Cif2VRML application [18].

The NIH 3D Print Exchange web tool, the Cif2VRML application, and the Jmol3DP script reported herein all have utility and unique benefits that help to advance the creation of 3D printable crystal structures. Looking toward the future, we reiterate, that the ideal solution to programmatically convert crystal structures into 3D printable files may be to produce separate 3D printable files for each molecule/extended solid in the CIF file so that no loss of structural representation occurs and users can select the structure of interest to 3D print.

**Fig. 3:**
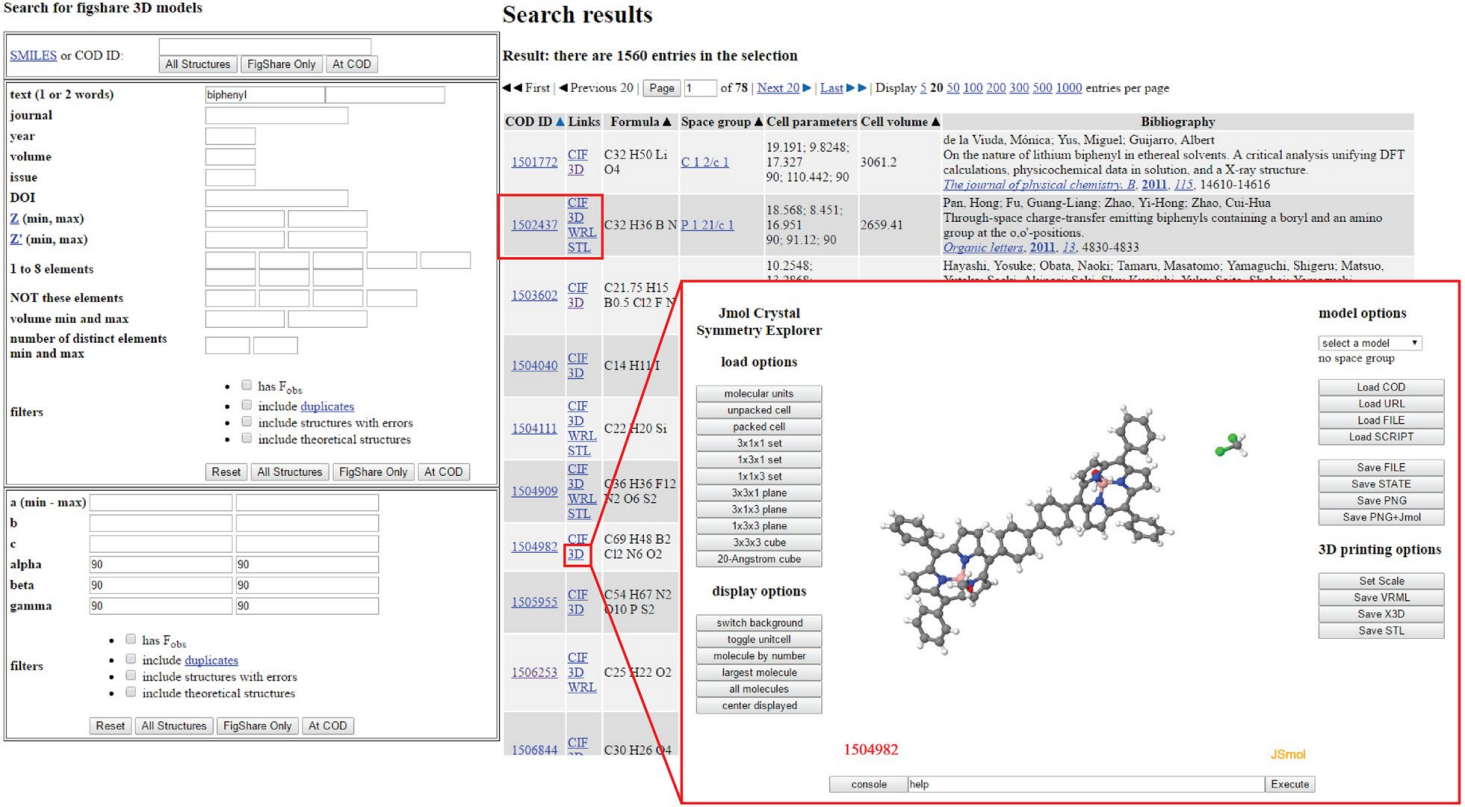
Example search for “biphenyl” on the Jmol 3D Print website: https://chemapps.stolaf.edu/jmol/3dprint. Users can discover over 30,000 VRML/STL 3D files through a modified version of the COD search interface. VRML and STL files are displayed and available for download alongside the COD entry record. The “3D” link next to each COD record directs users to a JSmol Crystal Symmetry Explorer website with options to create custom VRML and STL 3D printable files for any structure in the COD. User provided CIF data can also be readily converted into VRML and STL files

The available 3D printable crystal structures created in this work will allow researchers and educators to quickly locate a large collection (>30,000) of crystal structures that are 3D print ready; no further modifications to the crystal structure or file format should be needed. In addition to providing the entire collection on figshare, a Jmol 3D Print website was created that allows for discovery of the entire collection of 3D printable files created in this work. The STL and VRML files for the compounds were placed on the chemapps.stolaf.edu server in a set of zip files based on COD id 1000000-1999999, 2000000-2999999, etc. The list of ID numbers for all models was also included. The website works by making two calls to COD from the “Figshare Only” button. It first requests ID numbers for a user’s search involving SMILES, text, reference, elements, or cell parameters. The returned list of hits from COD is then filtered using the ID list for the 31,239 crystal structures, and this filtered list is sent back to COD, now requesting a full report for only these specific structures. Up to 100 structures are returned. The results are then displayed using a modified form of the COD search results page where users can download available VRML or STL files (Fig. 3).

If users wish to create a custom 3D printable file that is not available in the predefined file dataset created in this work, an option is available to search the entire COD and design custom 3D print files using JSmol, the non-Java HTML5 version of Jmol. When a search is executed on the Jmol 3D Print website using the “All Structures” button, hits from the COD are returned in a list with both the predefined VRML and STL files, if available, and a 3D link that opens a JSmol Crystal Symmetry Explorer website to allow custom creation of 3D print files. This JSmol Crystal Symmetry Explorer allows various symmetry load options, molecule selection options, and 3D printable STL and VRML file saving options. Moreover, users can even upload their own CIF data and scripts for custom conversion of their CIFs into 3D printable files (Fig. 3).

Creation of VRML and STL files within the JSmol Crystal Symmetry Explorer is a local operation executed in JavaScript within the web browser; there are no server processes necessary for preparing the 3D visualization and subsequently saving a VRML or STL file. As such, if users open the JSmol Crystal Symmetry Explorer from their local machine (from a copy of the JSmol distribution), CIF data can be readily processed into VRML and STL files without any connection to the internet. This process is in contrast to other web-based 3D crystal structure file converters such as the NIH 3D Print Exchange, which do this processing on a server and thus require an internet connection. Furthermore, since the capability of creating VRML and STL files is built into all versions of Jmol starting with version 14.6.4 (both Java and JavaScript), it will be possible to create 3D printable files of models displayed in Jmol at any website that has upgraded their implementation of Jmol to this version or higher (e.g. Crystallography Open Database, Protein Data Bank, Inorganic Crystal Structure Database, Cambridge Structural Database and more). To do so, users need only to click on the JSmol logo or right-click on the model window to call up a pop-up menu and select File > Export > Export VRML 3D Model or File > Export > Export STL 3D Model.

**Fig. 4:**
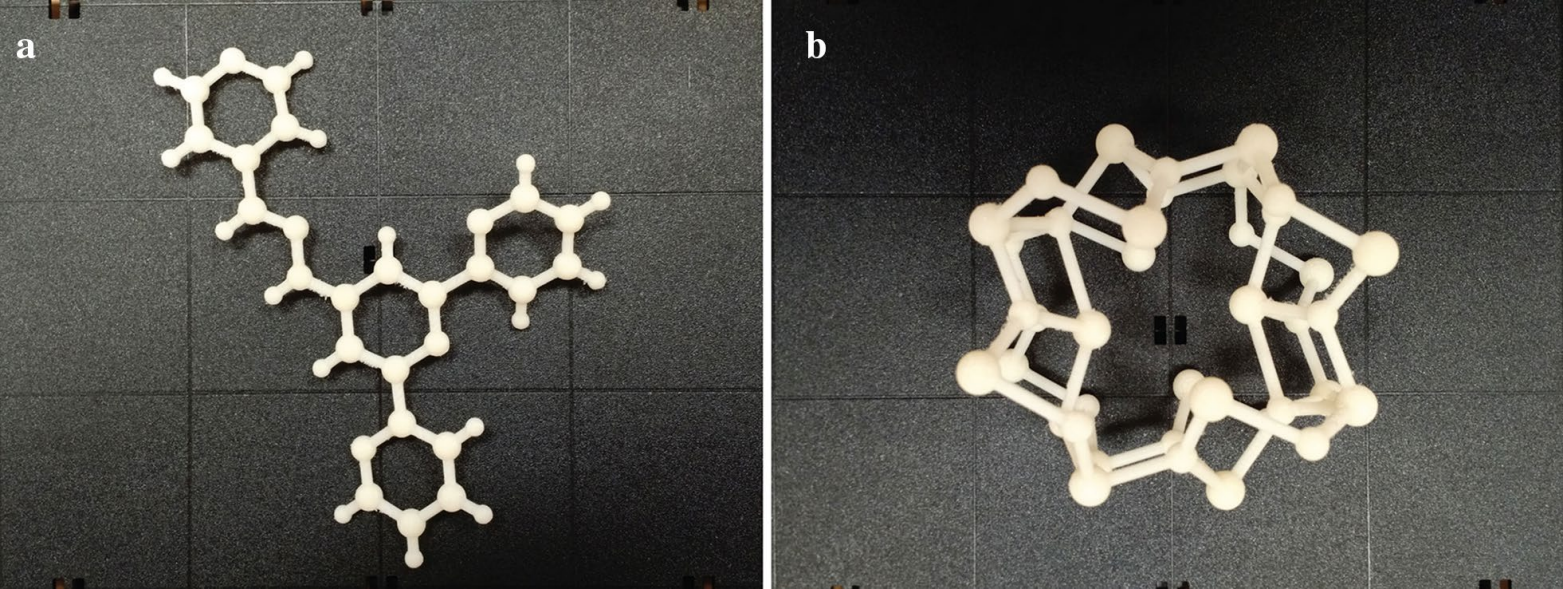
3D printed 4′-[(2E)-2-(4-Pyridinylmethylene)hydrazino]-2,2′:6′,2″-terpyridine (COD ID: 7004704) with approximate dimensions of 15 × 10 × 1 cm (**a**). 3D printed Pb_3_O_4_ (COD ID: 9013446) with approximate dimensions of 8 × 8 × 8 cm (**b**). Both models were 3D printed on a Stratasys uPrint SE using ABS P430 XL plastic

We have 3D printed several STL structures in-house from the produced dataset and a VRML file outsourced to Shapeways, confirming our programmatic methods successfully created 3D printable files (Fig. 4) [29]. Users can immediately load the 3D files into their 3D printing tool path software and begin fabricating the crystal models. Because many of the crystal models are complex, the use of a removable/dissolvable support material in the 3D printing process is recommended to achieve optimal results and quality of the printed model.

## Conclusions

Jmol scripting was used to programmatically convert 31,239 crystal structures (CIFs) from the COD into 3D printable files. These 3D printable files and Jmol scripts are available openly to the community on figshare and on a custom Jmol 3D Print website based on the COD search interface that allows users to discover the 3D printable crystal structure datasets via text, SMILES, reference, elements, or cell parameters. The Jmol 3D Print website also allows users to convert their own CIFs and structures in the COD (>350,000) into a 3D printable file using JSmol, the non-Java HTML5 version of Jmol. Our hope is that these 3D printable files will be deposited into popular databases such as PubChem [30, 31], ChemSpider [32, 33], and the Crystallography Open Database [13]. There is also future potential for implementing the JSmol Crystal Symmetry Explorer into the Crystallography Open Database [13] and other crystallography databases, allowing users to easily customize and create 3D printable crystal structure files. Current crystallography databases and websites that use Jmol can upgrade to v14.6.4 or later to allow users the option of exporting 3D printable VRML and STL files. We highly encourage the community to use the 3D printable crystal structure dataset, Jmol scripts, and Jmol 3D Print and Crystal Symmetry Explorer websites to advance their research, education, and outreach efforts with 3D printed molecular models.

## Abbreviations

3D: three-dimensional
CIF: crystallographic information file
STL: stereolithography file
VRML: virtual reality modeling language
COD: Crystallography Open Database
NIH: National Institutes of Health
